# Local shifts in floral biotic interactions in habitat edges and their effect on quantity and quality of plant offspring

**DOI:** 10.1093/aobpla/plx031

**Published:** 2017-07-26

**Authors:** Domenico Gargano, Giuseppe Fenu, Liliana Bernardo

**Affiliations:** 1 Dipartimento di Biologia, Ecologia e Scienze della Terra dell’Università della Calabria, Via P. Bucci, I-87036 Arcavacata di Rende, Italy; 2 Centro Conservazione Biodiversità (CCB), Dipartimento di Scienze della Vita e dell’Ambiente, Università degli Studi di Cagliari, Viale Sant’Ignazio da Laconi, 11–13, I-09123 Cagliari, Italy

**Keywords:** *Dianthus*, dichogamy, edge effect, inbreeding depression, plant mating systems, pollen limitation, self-fertilization

## Abstract

Spatial shifts in insect fauna due to ecological heterogeneity can severely constrain plant reproduction. Nonetheless, data showing effects of insect visit patterns and intensity of mutualistic and/or antagonistic plant–insect interactions on plant reproduction over structured ecological gradients remain scarce. We investigated how changes in flower-visitor abundance, identity and behaviour over a forest-open habitat gradient affect plant biotic interactions, and quantitative and qualitative fitness in the edge-specialist *Dianthus balbisii*. Composition and behaviour of the insects visiting flowers of *D. balbisii* strongly varied over the study gradient, influencing strength and patterns of plant biotic interactions (i.e. herbivory and pollination likelihood). Seed set comparison in free- and manually pollinated flowers suggested spatial variations in the extent of quantitative pollen limitation, which appeared more pronounced at the gradient extremes. Such variations were congruent to patterns of flower visit and plant biotic interactions. The analyses on seed and seedling viability evidenced that spatial variation in amount and type of pollinators, and frequency of herbivory affected qualitative fitness of *D. balbisii* by influencing selfing and outcrossing rates. Our work emphasizes the role of plant biotic interactions as a fine-scale mediator of plant fitness in ecotones, highlighting that optimal plant reproduction can take place into a restricted interval of the ecological gradients occurring at forest edges. Reducing the habitat complexity typical of such transition contexts can threat edge-adapted plants.

## Teaser

Habitat variations influence the richness and composition of insect guilds. This affects plant reproduction, which depends upon functional relationships with insects, responsible for both pollination and predation. Major consequences can occur in composite landscapes, where forest fragmentation produces transition habitats showing a great heterogeneity over short-spatial scales. We studied herbivory and pollination in an edge-specialist carnation over a forest-open habitat gradient. Visiting insects varied over the gradient, affecting herbivory and pollination rates, and offspring quality and quantity. Our findings emphasize the role of plant-insect interactions in tuning plant fitness in edge habitats, and provide guidelines for managing such ecological contexts.

## Introduction

Plant biotic interactions (i.e. plant functional relationships with mutualistic and antagonist organisms; *PBI*s hereafter) are a key driver of population processes in angiosperms, the largest group within the plant kingdom. In flowering plants, *PBI*s are mainly founded on associations with insects, which may act as pollinators or predators (Schoonhoven *et al.* 2005; Harder and Barrett 2006).

As far as mutualistic relationships are concerned, 308 006 flowering plant species, accounting for 87.5 % of the whole angiosperm diversity, show animal-mediated pollen transfer (Ollerton *et al.* 2011). The relevance of plant–pollinator interactions in preserving viable plant populations is emphasized by historical connections between insect and plant extinction patterns (Pauw and Hawkins 2011). Indeed, inadequate pollen delivery (i.e. pollen limitation) is recognized to limit plant fitness (Aguilar *et al.* 2006; Ghazoul 2005; Knight *et al.* 2005) and, then, to affect angiosperm evolution (Harder and Aizen 2010). Pollen limitation (*PL*) can derive either from quantitative or qualitative inadequateness of the pollen reaching the stigma (Wilcock and Neiland 2002). In the first case, *PL* originates from insufficient pollen transfer, which is typically due to the scarcity of pollinators (Ashman *et al.* 2004). Instead, qualitative *PL* is due to a poor quality of the pollen admixture deposited onto stigmas (Amat *et al.* 2011; Arceo-Gomez and Ashman 2014). Variations in abundance, composition and behaviour of pollinating fauna may represent a primary source of both quantitative and qualitative *PL* (Gómez *et al.* 2010). Such insect variations can rise from habitat changes (Totland 2001; Chalcoff *et al.* 2012), human disturbance (Brys and Jacquemyn 2012) and local structuring of plant populations (Zorn-Arnold and Howe 2007; Le Cadre *et al.* 2008).

Although much attention has traditionally paid to plant–pollinator relationships, recent work highlights the need of integrating studies on both plant–pollinator and plant–herbivore interactions in investigations on *PBI*s (Lucas-Barbosa 2016). Herbivory often exerts major constraints on plant reproduction (Cariveau *et al.* 2004; Ashman and Penet 2007). For instance, florivory may alter the efficiency of relationships between plants and pollinators. This may increase *PL* in plant populations, due to the reduced pollen transfer caused by pollen consumption (Hargreaves *et al.* 2009; Harder and Aizen 2010), and to herbivory-induced variations in plant traits involved in pollinator attraction, like floral display (Penet *et al.* 2009; Sõber *et al.* 2010) and scent (Lucas-Barbosa *et al.* 2011, 2016). Such interplay between mutualistic and antagonistic relationships has also important evolutionary implications, often resulting in selection conflicts on traits mediating insect attraction or avoidance (Gómez 2003, 2005).

Because habitat variations influence patterns of insect presence, abundance and behaviour (Totland 2001; Caruso *et al.* 2003), plant processes relying on *PBI*s can show relevant spatial variations (Gómez *et al.* 2010). These variations may be particularly evident in proximity of habitat edges. Indeed, the switch between different habitats generates transition zones with emergent ecological properties recognized as ecotones (Risser 1995). In landscapes subjected to a long-lasting human impact, most of spatial ecological heterogeneity (and ecotones) derives from the alteration of pristine habitats like forests. This contributes to form a kaleidoscope of transition habitats, which change in space and time depending on local patterns of disturbance and vegetation dynamics (Dovčiak and Brown 2014). Studying ecological responses at forest edges (i.e. edge effect) is a key topic in landscape ecology and biodiversity conservation (Forman 1995; López-Barrera *et al.* 2007; Hadley and Betts 2012). Indeed, understanding how ecological processes vary near forest edges is crucial to evaluate how landscape-level dynamics, such as fragmentation, affect biodiversity (Ries et al. 2004). Overall, species interactions are retained one of the main mechanisms influencing biodiversity patterns in habitat edges (Ries et al. 2004). Accordingly, plant population processes depending on *PBI*s (i.e. herbivory, pollination and dispersal) are influenced by the proximity to forest edges (Chacón and Armesto 2006; López-Barrera *et al.* 2007). The extent of such kind of edge effect depends on local levels of ecological heterogeneity (Sarlov-Herlin 2001; López-Barrera *et al.* 2007), and disturbance (Bhattacharya *et al.* 2003; Huang *et al.* 2009). Because the functioning of *PBI*s is influenced by the size and spatial configuration of habitat patches suitable for plant species (Lennartsson 2002; Verboven *et al.* 2014), the influence of habitat heterogeneity on *PBI*s becomes more relevant in highly fragmented landscapes (Hadley and Betts 2012).

Studying *PBI*s patterns in the proximity of forest edges would improve plant conservation in complex ecological mosaics (Hadley and Betts 2012), as those found in the Mediterranean region. In this area, due to the fragmentation of natural habitats, habitat edges occupy a conspicuous portion of current landscapes, and are expected to augment in the future, especially in lowlands (Falcucci et al. 2007). Consequently, such contexts will represent the theatre for evolutionary and conservation challenges of an increasing number of plants. Nonetheless, the management requirements of such anthropogenic ecotones are often neglected. To date, there is a substantial scarcity of field research investigating how small-scale habitat variations affect *PBI*s (including plant–pollinator and plant–herbivore interactions), and then plant reproductive performance along structured ecotone gradients.

We evaluated the influence of ecological heterogeneity on *PBI*-driven population processes in *Dianthus balbisii* Ser., an edge-specialist inhabiting ecotones between woody and open habitats in Mediterranean low lands. Field work aimed to (i) identify potential pollinators and herbivores of *D. balbisii*; (ii) evaluate the aptitude of different pollinators versus intra- or inter-plant visits; (iii) check for visit frequency variations along a gradient of light intensity (assumed as a proxy of habitat structure); (iv) perform floral manipulations to compare the outcome of natural pollinations with that of forced crossing and forced selfing along the studied ecological gradient. Such data were used to ask the following questions: (i) How did insect patterns change along the studied gradient? (ii) Did variations of insect visit patterns cause functional shifts in *PBI*s (i.e. pollination likelihood, herbivory rate, amount and quality of pollen transfer)? (iii) Did such shift in *PBI*s influence quantitative and qualitative components of plant reproductive fitness along the studied ecological gradient?

By answering such questions, we would provide basic insights on the relationships between *PBI*s and plant reproductive fitness in anthropogenic ecotones, an integral component of complex landscapes.

## Methods

### Study species and study area

The perennial *Dianthus balbisii* Ser. has a woody stock bearing up to 20 flowering stems ending with a multi-flowered head (2–20 flowers × head); the corolla is reddish, by distinguishing *D. balbisii* from the closely related *D. guliae* Janka (Peruzzi and Gargano 2006). The flowering season is from late May to late June. The distribution range of *D. balbisii* embraces the Central Mediterranean area, where this species occurs in hilly areas at the border between woody and open xeric communities (Pignatti 1982). As in several congeneric species (Erhardt 1988), the plant is self-compatible, and shows a protandrous hermaphroditism with the possible occurrence of female flowers (i.e. gynomonoicism and gynodiocism). *Dianthus* species have rather specialized pollination syndromes, involving Lepidoptera as main pollinators (Erhardt 1988). Floral traits and architecture (i.e. reddish flowers with short calix grouped in dense heads) suggest that *D. balbisii* belongs to the carnations pollinated by diurnal butterflies (Erhardt 1988; Block *et al.* 2006). Field observations (i.e. daily flower monitoring) also support that in this species most of pollinations occur during the day (D. Gargano pers. obs.). In the study area (Calabria, Southern Italy, N 39°33′; E 16°22′), *D. balbisii* occurs near the border of forests dominated by deciduous oaks, and in adjacent garrigue-like communities.

### Sampling individuals and structuring the habitat gradient

The overall study population accounted approximatively for 150 plants; these were rather uniformly distributed over a forest ecotone (∼150 × 50 m) encompassing two adjacent sites in a highly anthropized area. In spring 2014, 83 individuals of *D. balbisii* were permanently marked before flowering by selecting plants that were at least 5 m apart. From the whole sample, 40 plants were used to collect data about pollination and herbivory. The remaining 43 plants were employed in experimental pollinations. In this way, possible interference of floral manipulation procedures (e.g. bagging) with *PBI*s were avoided.

The ecotone structure was determined based on measures of canopy closure. This parameter was chosen because variations in habitat canopy are recognized to influence both abiotic (i.e. temperature and soil moisture) (Joffre and Rambal 1993) and biotic (Totland 2001; Chacόn and Armesto 2006; Lόpez-Barrera *et al.* 2007) drivers of plant fitness. The position over the ecotone of each sampled plant was determined by using light intensity (*LI*) as a proxy of habitat canopy closure. A direct measure of incident light was chosen because it can be effective in recording small-scale canopy variations near the ground surface, as congruent with the size of the study species. Direct solar radiation measures can also help in interpreting patterns of pollinator activity (Kastinger and Weber 2001). Moreover, this method allows many replications to obtain more stable average values. *LI* was measured on the surface of the flower heads by a luxometer (Panlux electronic 2, Gossen, Nürnberg). For plants that had up to three flowering stems the light intensity was measured on all stems, and the resulting values were averaged. Instead, for plants with many flowering stems, three measures were taken on the three tallest stems, and the recorded values were then averaged. All measures were carried out after the canopy of deciduous trees completed its development. To reduce the effect due to the moment in which they were taken, all the measurements were repeated during two consecutive sunny days (12–13 June) from 12.00 a.m. to 2.00 p.m.

*LI* measurements were used to structure a gradient of light intensity encompassing five classes hosting a comparable amount of sampled individuals: (i) *LI* < 500 Klux (No. plants = 16), (ii) 500 < *LI* < 1500 Klux (No. plants = 17), (iii) 1500 < *LI* < 2500 Klux (No. plants = 17), (iv) 2500 < *LI* < 4000 Klux (No. plants = 17), (v) *LI* > 4000 Klux (No. plants = 16). Because canopy variations regulate spatial resource patterns in temperate habitats (Joffre and Rambal 1993), plants occurring in the same *LI* interval were assumed to have a comparable amount of available resources as they occurred under similar canopy conditions.

### Visiting insects

Individual patch size (no. of stems, no. of buds per stem and per plant) was estimated for the 40 plants (8 × LI class) selected to monitor insect visits.

Observations on insects visiting *D. balbisii* were carried out over four consecutive days (from 12 June to 16 June) in the middle of the species’ blooming season. Overall, they accounted for 560 min of direct field observations for recording each insect visit on each marked plant. Such observations were organized in trials of 20 min, which were carried out during fixed intervals in the morning (from 10.30 a.m. to 12.30 a.m.) and in the afternoon (from 3.00 p.m. to 5.00 p.m.). Plants were subjected to 28 observation runs (16 in the morning and 12 in the afternoon). To minimize disturbance to insects, the observers remained seated some meter apart the plants. Due to the gradual blooming typical of the study species, each observation trial was preceded by the annotation of the amount of flowers opened on the plants. This allowed us to relate visit patterns to the effective flower display (EFD = no. of opened flowers on the plant at the visit time).

When possible, the identity of insect taxa was assessed in the field. Unknown insects visiting *D. balbisii* flowers were identified in laboratory with the help of expert entomologists based on images and specimens collected in the field. Taxa unidentified at the specific level were assigned to higher taxonomic ranks (i.e. genus and family). The observed insects were categorized in four functional categories. Firstly, they were distinguished into pollinators or herbivores based on literature knowledge and field observations. Butterflies were always considered as pollinators as they are the main pollinators of *Dianthus* species (Erhardt 1988). Also *Bombylius major* L. (Diptera), that frequently visited flowers of *D. balbisii*, was included among pollinators. Indeed, bee-flies are recognized as pollinators in many plant species with tubular flowers (Kastinger and Weber 2001). Then, the primary pollinators (the lepidopteran *Thymelicus sylvestris* Poda and *B. major*) were separated from secondary pollinators (other lepidopterans, OTH) based on visit frequency. Furthermore, *T. sylvestris* and *B. major* were assigned to two distinct categories (named, respectively, THY and BOM) because of relevant functional differences (i.e. diverse preferences with respect to the investigated gradient, diverse aptitude to within- and among-plant visit). Finally, weevils and ants were classified as florivores (McCall and Irwin 2006; Penet *et al.* 2009) or pollen and nectar robbers (Soper Gorden and Adler 2016), respectively. Since weevils were widely dominant, all florivores were included into a single category (HER). HER also included pre-dispersal predators that were identified in laboratory based on the caterpillars found in fruits of *D. balbisii* collected in the field. Joining florivores and pre-dispersal predators into a single unit was justified by the very low amount of seeds produced by flowers that experienced florivory (see below).

To estimate the potential for cross- and self-fertilization of each visiting taxon based on its behavioural aptitude, visits were classified as (C) (visits among individuals, potentially allowing for cross-pollination), or (G) (visits within individual, potentially allowing for geitonogamy). The (C) tag was used when insects visited an individual of *D. balbisii* and then moved away. Differently, the (G) tag applied to visits of insects coming from other flowers of the same plant (in other words, multiple consecutive visits on a same plant qualified as G).

### Flowering regime

The 40 plants were monitored daily over 3 weeks (from 14 June to 5 July), by annotating amount and sex of open flowers. Since the species is protandrous, the hermaphrodite flowers were distinguished into males (i.e. flowers bearing only emergent stamens), and hermaphrodites (i.e. flowers bearing both stamens and stigma branches). Finally, male-sterile flowers (i.e. bearing only stigma branches) were classified as female. Flower sex data were used: (i) to quantify the frequency of male-sterility within the population, and (ii) to estimate pollination frequency over the study gradient (i.e. in proterandrous species, large accumulations of flowers in the hermaphrodite stage can indicate slow pollination processes).

During such field trials we also recorded flowers that resulted pollinated (i.e. flowers with closed corolla) or showed signs of florivory (i.e. petals, stamens and/or stigma branches eaten by herbivores). This work consisted in 2737 field observations involving 656 flowers.

### Floral manipulations

Fertility of perennial plants can be affected by several maternal traits (e.g. age, size, history, genetics and environmental conditions). This can bias fitness comparisons among pollination treatments carried out on different individuals. To reduce such bias, floral manipulations were designed to obtain matched offspring for various pollination protocols (see below) from each experimental plant. To this end, from the initial 43 individuals, plants bearing <5 flowers were not manipulated, and the final amount of treated plants became 30 (6 × *LI* class).

The pollination experiments performed on these plants involved at least one flower for each of four treatments: forced crossing (No. = 80), forced selfing (No. = 76), autonomous selfing (No. = 74) and free pollination (No. = 80). Except for the sample used for free pollination, the flowers were isolated at the early bud stage by bagging 1–2 inflorescences per plant. In flowers used for forced cross-pollination, anthers were gently removed with a dissection forceps before the emergence of stigma to avoid occasional selfing. Hand-pollinations (i.e. forced crossing and forced selfing) were carried out by brushing one dehiscent anther per stigma branch; this would allow for fertilizing all ovules (<100) usually found in the ovaries of *D. balbisii*. The stigma was retained receptive when it was well developed and appeared glutinous and papillose. Self-pollen was taken from other flowers of the same plant. Pollen used in forced-crossing was taken from >30 plants, each distant >5 m from the recipient plant. Occurrence and extent of autonomous self-pollination was evaluated by leaving untreated 1–2 bagged flowers per plant. As the last flower in the head closed petals or withered, the bag was removed to allow fruit development under natural conditions.

### Fitness components

All flowers left free were used to investigate rates of herbivory, pollination and seed set under natural pollination regime. At the end of the blooming season all fruits occurring on the marked plants were collected. In the laboratory, fruits were checked for occurrence of pre-dispersal predation (e.g. presence of caterpillars, remnants of seeds eaten by herbivores). In these way fruits were distinguished into three categories: predated, aborted and fertilized. This screening accounted for 1185 fruits from 40 plants. Except for three cases (on 571), no seeds were produced by flowers that experienced florivory or pre-dispersal predation. Therefore, we annotated for each flower the occurrence of herbivory or pollination, and in the latter case the number of seeds.

The seeds contained in each fruit were counted and, then, a sample of 450 seeds was germinated to assess seed viability and early seedling survival rate. At the beginning of the autumn seeds were sowed on wet paper in Petri dishes at 18 °C. The resulting seedlings were transferred into 4 × 4 cm pots containing a mixture of peat moss and compost with presence of pumice fragments. Subsequently, 30-day old seedlings were transplanted into 20 × 17 cm pots filled with brown soil and subjected to periodical irrigation and fertilization (20 ml of a mixture of ammonium (6 %), phosphorous (5 %), and potassium (7 %) diluted in H2O was monthly supplied). The plants were maintained in glasshouse for 6 months at Botanic Garden of the Università della Calabria.

### Data analysis

The efficacy of the sampling effort in capturing information on insects visiting *D. balbisii* was evaluated by a rarefaction procedure on sample-based abundance data (Colwell *et al.* 2012). To this end it was applied the rarefaction function provided by EstimateS 9.1.0 (Colwell 2013).

The relationships between visit frequency (no. of visits × plant patch × observation run) and *EFD* were checked by a Pearson correlation test. Variations of visit frequency across levels of light intensity were checked by a Generalized Linear Model (GLM), by considering the *LI* class as fixed factor and *EFD* as covariate. The association between visitor type and light intensity, and between visitor type and visit type was evaluated by cross-tabulation analyses using *chi*-square as a measure of association.

Variations of individual patch size (i.e. no. of stems per plant, no. of buds × per plant and no. of buds per stem) across intervals of light intensity were analysed by one-way Anova. Patterns of sex ratio were evaluated by calculating from daily data the individual hermaphroditic rate (no. of hermaphrodite flowers/total no. of open flowers per plant) and the individual female rate (no. of female flowers/total no. of open flowers per plant). Then, differences among intervals of light intensity in average individual hermaphroditic and female rates were checked by one-way Anova.

The strength of *PBI*s was evaluated based on the variations of herbivory, pollination chance and seed set over the five *LI* classes. In the analyses, herbivory accounted for both florivory and pre-dispersal predation, because they had an equivalent effect of the reproduction of *D. balbisii* (i.e. both caused absence of seed-set). To include the effect of plant traits involved in insect attraction on herbivory and pollination likelihood, we comprised in such analyses the inflorescence size (i.e. the amount of flowers occurring in the same head of each studied fruit). Herbivory was a binary variable indicating presence or absence of florivory and pre-dispersal predation. The pollination chance also consisted in binary data indicating occurrence or absence (caused by lack of visits, herbivory or abortion) of fertilization. Variations of herbivory and pollination chance among *LI* classes were then analysed by GLM, including the *LI* class as fixed factor and the inflorescence size as covariate. Quantitative fitness analyses were based on seed set (i.e. no. of seeds per fruit) comparisons between naturally pollinated and manipulated plants. Flowers that were not pollinated were considered by assigning a seed set = 0. Seed set in not-predated flowers (570 flowers from 40 plants) was analysed by one-way Anova to estimate seed set variations among the 5 *LI* classes. To evaluate the effect of qualitative pollen limitation on seed production, a GLM was used to analyse the seed set of 75 fruits per each of three treatments: forced-crossing, forced selfing, natural pollination (3 fruits × 5 plants × 5 *LI* classes × 3 treatments) using *LI* class and pollination treatment as fixed factors.

Further qualitative fitness components (i.e. seed germination and survival in 6-month-old seedlings) were estimated by considering the sample of 450 seeds (6 seeds × 5 plants × 5 *LI* classes × 3 pollination treatments) obtained by plants under free-pollination, forced crossing and forced selfing. Autonomous self-fertilization was not considered because it was ineffective in most of cases. Seed germination and seedling survival were treated as binary variables, and were analysed by GLM (using *LI* interval and pollination treatment as fixed factors). GLM analyses on binary variables were performed by using the LOGIT link function. Response variables accounting for count data (i.e. visit frequency, no. seeds × fruit) were not normally distributed. Therefore, in GLM these variables were analysed by using a LOG link function, which is appropriate for data represented by non-negative integer values. Because floral manipulations (i.e. pollen supplementation) can promote resource reallocation within plants, this may result in a higher pollen limitation in free-pollinated flowers of manipulated plants (Knight *et al.* 2006; Wesselingh 2007). The possible effects of such reallocation processes were tested by a *t*-test on independent samples on the seed set of 160 free pollinated flowers, 80 were randomly selected from the 30 manipulated plants, and 80 from 30 non-manipulated individuals within the same *LI* interval.

All these analyses were performed in SPSS 24 for Windows (SPSS, Chicago, IL, USA).

## Results

### Patterns of visit abundance, visitor type and behaviour over the studied gradient (question 1)

**Figure 1.**
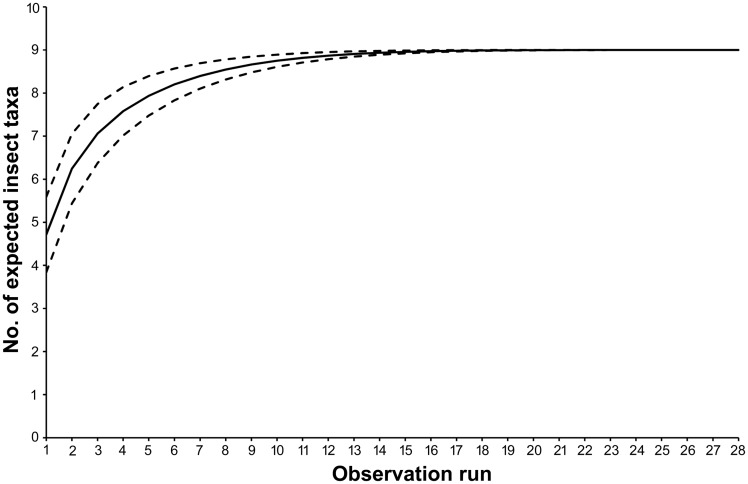
Species accumulation curve showing the expected no. of insect taxa as a function of the observation effort (no. of observation runs). Dashed lines delimitate the 95 % confidence intervals of estimations.

In average, we recorded 4.7 ± 1.9 insect taxa per observation run. The rarefaction function suggested that the observation effort provided a reliable estimation of the insect fauna interacting with the flowers of *D. balbisii* (Fig. 1).

**Figure 2.**
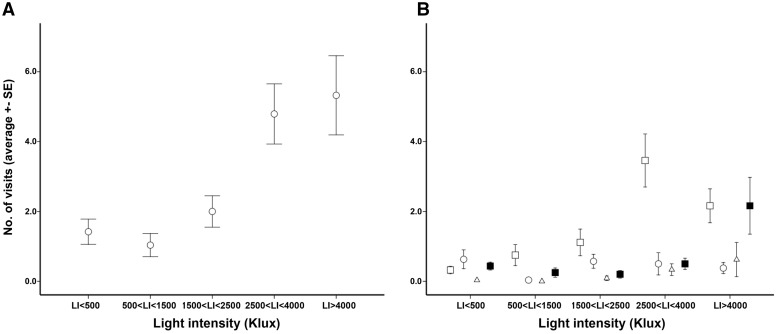
Patterns of insect visit over the study habitat gradient: overall visit frequency (**A**), and visit frequency of the different insect types (**B**) across classes of light intensity [white squares = *Thymelicus sylvestris* (THY); white circles = *Bombylius major* (BOM); white triangles = other putative pollinators (OTH); black squares = herbivores (HER)].

GLM analyses indicated that light intensity influenced visit frequency (Wald *chi*^2^ = 29.46, *P < *0.001). More than 71 % of documented visits occurred under a light intensity exceeding 2500 Klux (Fig. 2A). The effective floral display (*EFD*) also significantly influenced the visit frequency (Wald *chi*^2^ = 24.05, *P < *0.001). Accordingly, visit frequency was significantly related to *EFD* (*r* = 0.389; *P < *0.001; No. = 384). However, the significant interaction between *LI* and *EFD* (Wald *chi*^2^ = 15.89, *P = *0.003) suggested that the effect of *EFD* on visit frequency varied along the ecological gradient.

The list and frequency of recognized insects, along with their putative functional role, is provided in Table S1 [**see Supporting Information**]. Among pollinators, *T. sylvestris* (THY) was responsible for 53.2 % of visits, followed by *B. major* (BOM = 16.9 %), and a mixture of other butterflies (OTH = 5.6 %). The remaining visits were due to herbivores (HER = 24.3 %). Among the herbivores, florivores (85.5 %) were much more frequent than pollen/nectar eaters (14.5 %) [**see****Supporting Information**—**Table S1**]. The frequency of the different visitors varied among intervals of light intensity (Wald *chi*^2^ = 86.389, *P < *0.001). The relative visit frequency of THY ranged between 0.28 (*LI* < 1000 Klux) and 0.73 (2500 < *LI* < 3500 Klux), and then decreased at higher light intensities (Fig. 2B). Instead, BOM was the most frequent visitor in the lowest *LI* class (Fig. 2B). The proportion of visits due to HER varied from 0.08 to 0.36, with lower frequencies at intermediate *LI* intervals (Fig. 2B). The remaining putative pollinators had much lower visit frequency (<0.1 in most *LI* intervals) (Fig. 2B).

Different visitors were significantly associated to different movement patterns (i.e. among- and within-plant) (Wald *chi*^2^ = 18.34, *P < *0.001). Most of within-plant movements were due to BOM (50.7 %) and HER (69.7 %). Instead, movements among plants prevailed in OTH (60.9 %) and, especially, in THY (76.0 %).

### Relating insect visit and flowering regime to patterns of *PBI*s (question 2)

**Figure 3.**
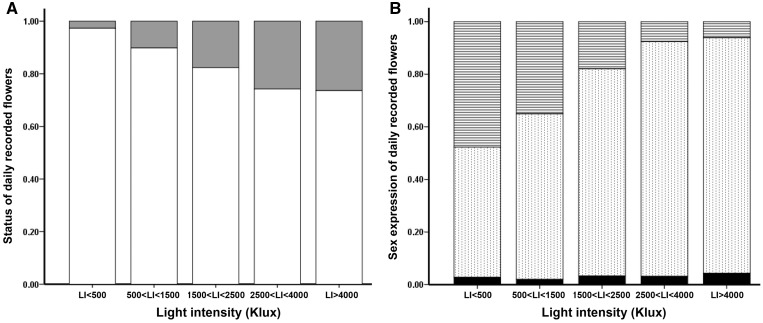
Status and sex ratio of the flowers daily recorded over the gradient of light intensity. (**A**) Relative proportion of open flowers (white bar) and of flowers pollinated or damaged by herbivory (grey bar). (**B**) Proportion of flowers found in hermaphroditic (stripped bar) or male (dotted bar) stage, and fraction of female flowers (black bar).

Patch size traits differed among intervals of light intensity (Table 1). The no. of buds per plant (*F* = 4.948, *P* < 0.001; *N* = 40), the no. of stems per plant (*F* = 3.454, *P* = 0.02, *N* = 40), and the no. of buds per stem (*F* = 4.321, *P* < 0.001, *N* = 40) significantly varied over the gradient, reaching higher values in sunny places. The average individual amount of open flowers found in daily recognitions decreased toward high *LI* intervals (Fig. 3A), while the rate of florivory showed an opposite patterns (Fig. 3A). Daily average values of individual hermaphroditic rate strongly differed over the *LI* gradient (*F* = 15.117, *P* < 0.001, *N* = 40), revealing larger scores at lower *LI* (Fig. 3B). This suggested that, at lower *LI*, flowers required a longer time to be visited, as congruent with general patterns of insect visit. On the contrary, individual female rate (Fig. 3B) did not reveal significant patterns (*F* = 0.167, *P* = 0.955, *N* = 40).

**Table 1.** Average values (±SD) of patch size and effective floral display (no. of opened flowers on a plant at the time of insect visit) across classes of light intensity (*LI*). *EFD*, effective floral display.

*LI* class	Stems × plant	Buds × plant	Buds × stem	*EFD*
*LI* < 500 Klx	5.30 ± 3.6	28.50 ± 26.4	4.90 ± 2.2	7.57 ± 3.8
500 < *LI* < 1500 Klx	5.75 ± 3.3	37.50 ± 31.8	5.75 ± 2.5	9.08 ± 5.1
1500 < *LI*< 2500 Klx	5.67 ± 5.7	43.67 ± 33.3	11.94 ± 8.7	20.29 ± 13.4
2500 < *LI* < 4000 Klx	6.50 ± 7.3	50.50 ± 48.9	9.05 ± 6.3	12.13 ± 8.2
*LI* > 4000 Klx	11.60 ± 11.3	61.80 ± 57.3	5.80 ± 0.8	7.44 ± 3.4

**Figure 4.**
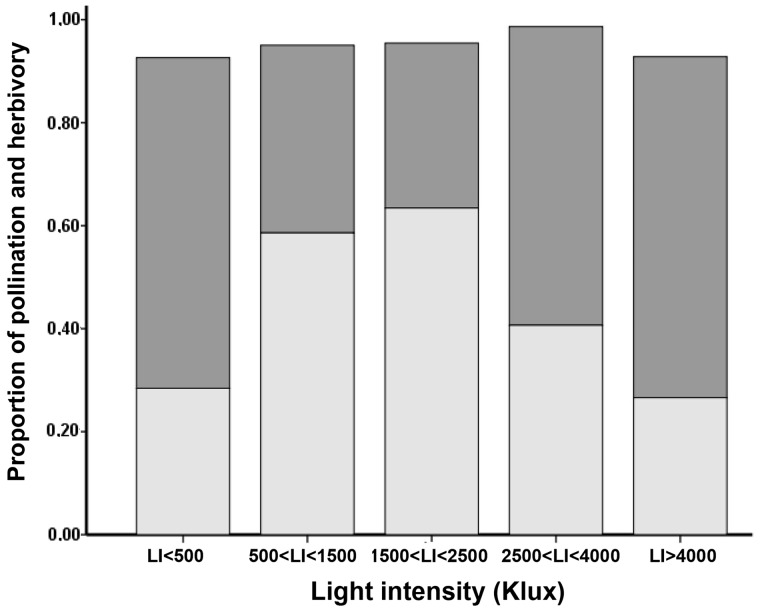
Relative proportion of pollination and herbivory over the gradient of light intensity. Light grey bar, proportion of pollinated flowers. Dark grey bar, proportion of flowers subjected to herbivory.

A variation of *PBI*s along the studied gradient was confirmed by GLM. Indeed, light intensity, inflorescence size and their interaction) had significant effects on herbivory and pollination chance (Table 2). Pollination increased from low to intermediate intervals of light intensity and then decreased at higher *LI*, while herbivory showed an opposite pattern (Fig. 4).

**Table 2.** Effect of light intensity (*LI*) and inflorescence size (*IS*) on likelihood of herbivory and pollination.

Source of variation	Dependent variable	Wald *chi^2^*	*P*

*LI*
	Herbivory	40.956	<0.001
	Pollination	37.540	<0.001
*IS*			
	Herbivory	35.785	<0.001
	Pollination	26.916	<0.001
*LI* × *IS*			
	Herbivory	13.977	0.007
	Pollination	10.000	0.04

### Quantitative and qualitative consequences on plant fitness (question 3)

**Figure 5.**
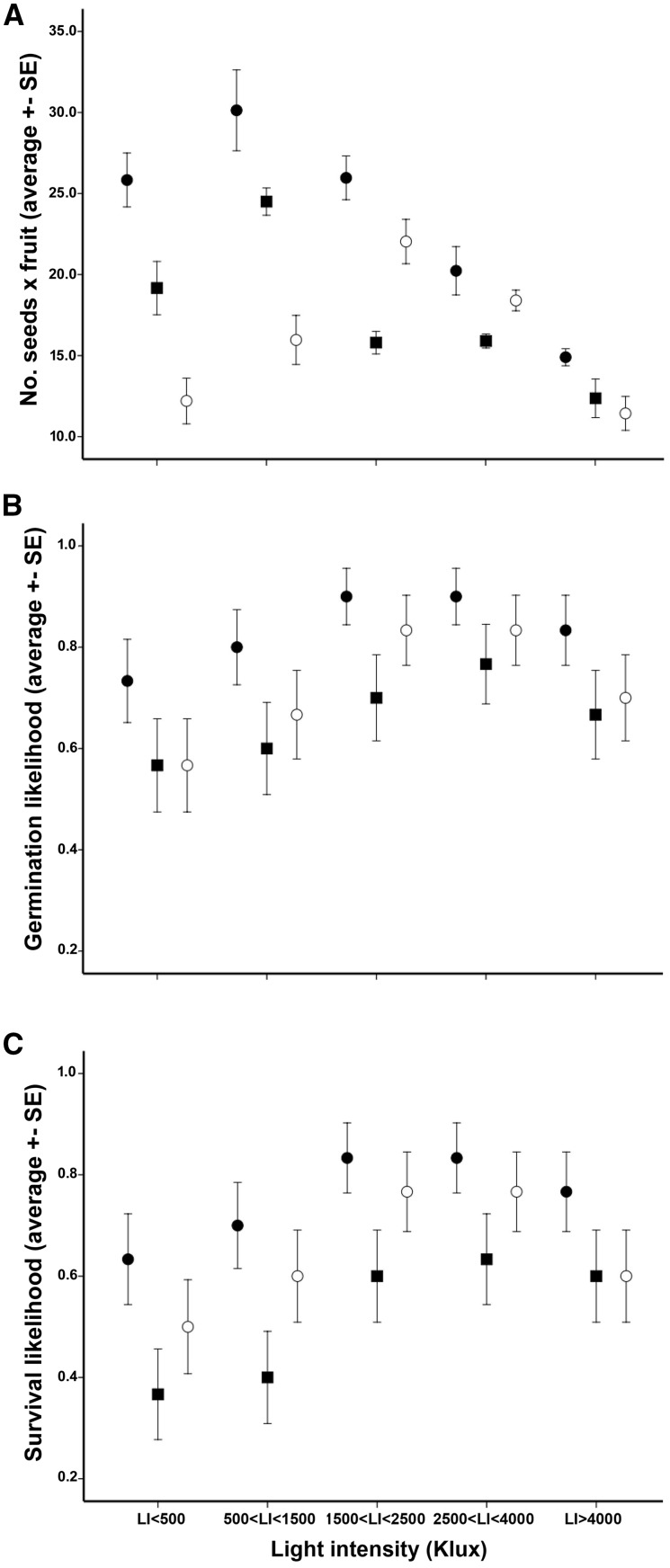
Fitness traits patterns across intervals of light intensity and pollination treatments: no. of seeds per fruit (**A**), seed viability (**B**) and seedling survival (**C**). Solid circles, hand-crosspollination; solid squares, forced-selfpollination; empty circles, free-pollination.

The no. of seeds per fruit varied significantly over the gradient (*F* = 14.439, *P* < 0.001), showing a decline in higher *L*I intervals (Fig. 5A).

Among manipulated plants, 25 individuals (5 × *LI* class) produced matched fruits for forced cross-pollination, forced self-pollination and free-pollination. Instead, many flowers and plants failed to produce seeds by autonomous selfing (<14 % of flowers produced fruits containing in average 2.45 ± 6.88 seeds). The seed-set of free-pollinated flowers on non-manipulated plants did not differ from free-pollinated flowers on plants subjected to experimental pollinations (*t* = 1.542, *df *=* *160, *P *= 0.098). The two way ANOVA on manipulated flowers evidenced substantial differences in seed set among forced selfing, forced crossing and free pollination (Table 3). The lower seed set was found in flowers left to free pollination. In 14 % of cases they did not set fruits, and their average seed set (14.75 ± 7.78) was lower than those found in flowers treated with forced selfing (18.05 ± 6.56) and forced crossing (23.41 ± 10.29). *LI* had significant effect on seed set patterns also in experimentally pollinated flowers (Table 3). The no. of seeds obtained by free pollination was lower than in flowers treated with forced crossing (average difference = −0 8.66, *P* < 0.001, *N* = 150) and forced selfing (average difference = −0 2.79, *P* = 0.003, *N* = 150). As shown in Fig. 5A, seed set from free pollination overcame forced selfing in two *LI* intervals (*LI*3: difference = 6.23, *P* < 0.001, *N*  =  30; *LI*4: difference = 3.21, *P*  =  0.03, *N* = 30). In the same *LI* intervals, the difference between free pollination and hand cross-pollination remained significant but it was less pronounced (*LI*3: difference = 3.93, *P* = 0.046, *N* = 30; *LI*4: difference = 3.70, *P* = 0.015, *N* = 30).

**Table 3.** Effect of light intensity and pollination treatment on the seed set of manipulated flowers.

Source of variation	*F*	*P*
Light intensity	26.489	<0.001
Pollination treatment	55.292	<0.001
Light intensity × Pollination treatment	8.164	<0.001

*LI* and pollination treatment influenced both germination and survival (Table 4). Differences in survival were higher than variations in germination (Fig. 5B, C). Overall, germination rate in cross-pollinated flowers was significantly higher to both free pollinated (83.3 % vs. 72.0 %, *P* = 0.024) and self-pollinated ones (83.3 % vs. 66.0 %, *P* = 0.001). On the contrary, the difference between free pollination and forced selfing was not statistically significant (*P* = 0.231). Seedling survival revealed a similar pattern, although the amount of survived plants was generally lower compared to germination rates (cross-pollination = 75.3 %; free-pollination = 64.7 %; self-pollination = 52.0 %). The differences between cross-pollination and forced selfing (*P* < 0.001) were higher than between cross-pollination and free pollination (*P* = 0.048). Instead, differences between free pollination and forced self-pollination were marginally significant (*P* = 0.051). At intermediate *LI* intervals (LI = 3–4), fitness differences between cross- and free-pollinations were not significant (*P* = 0.456 and *P* = 0.527 for germination and survival, respectively) (Fig. 5B, C). Instead, at highest *LI* the fitness related to free pollination approached that of self-pollination for both germination (*P* = 0.229) and survival (*P* = 0.171) (Fig. 5B, C).

**Table 4.** Influence of light intensity and pollination treatment on seed germination and seedling survival.

Source of variation	Dependent variable	Wald *chi^2^*	*P*
Light intensity
	Seed germination	13.860	0.008
	Seedling survival	17.487	0.002
Pollination treatment			
	Seed germination	11.756	0.003
	Seedling survival	17.637	<0.001
Light intensity × Pollination treatment			
	Seed germination	1.076	0.998
	Seedling survival	1.551	0.992

## Discussion

### Linking ecological heterogeneity to patterns of visit frequency and type (question 1)

Spatial ecological heterogeneity can promote substantial small-scale effects on insect webs (Totland 2001; Caruso *et al.* 2003). Accordingly, our data evidenced substantial variation in the overall and relative frequency of insect visits over the investigated ecological gradient. The individuals of *D. balbisii* occurring in sunny places experienced high visitation rate, confirming the general expectation that, in forest-edge contexts, insects prefer the more productive open patches (Chacón and Armesto 2006). However, we also recorded relevant variation in the relative visit rate of different insects over the investigated gradient. Substantially, this resulted in three major patterns: (i) infrequent visits that were mainly due to *Bombylius* flies at low *LI*, (ii) high visitation rate, prevalently due to butterflies, at intermediate *LI* and (iii) increasing visits of florivores at higher *LI*.

### Linking ecological heterogeneity and *PBI*s patterns (question 2)

The shifts in the visitation rate of mutualistic and antagonist insects influenced strength and patterns of *PBI*s over the *LI* gradient. Indeed, the individual patterns of flower accumulation showed a lower visitation rate at low *LI*. Instead, a prevalence of mutualistic (i.e. pollination) and antagonist (i.e. herbivory) interactions occurred at intermediate and high *LI*, respectively. Because the interplay between mutualistic and antagonistic interactions can generate selection conflicts on plant traits involved in insect attraction (Gómez 2003, 2008), such patterns of insect preferences could have affected the relationships between floral display size and pollinators. Indeed, although larger plant patches occurred at higher *LI*, here herbivory overcame pollination (Fig. 4). The frequency of herbivores at high *LI*, and their aptitude to perform multiple within-plant visits may have constrained pollination in many-flowered plants by reducing the available flowers, and by limiting the plant ability in attracting pollinators. Accordingly, herbivory was often found to alter plant traits involved in pollinator attraction, like scent (Lucas-Barbosa *et al.* 2011, 2016) and floral display (Penet *et al.* 2009; Sõber *et al.* 2010). Such findings agree with a non-additive pattern between positive and negative interactions, highlighting that a large floral display size can become disadvantageous in antagonist-rich contexts (Gómez 2003, 2005). 

### Linking ecological heterogeneity, *PBI*s and plant fitness (question 3)

Hand-pollinations may bias evaluation of pollen limitation, by promoting resource reallocation between non-manipulated and manipulated flowers of treated plants (Knight *et al.* 2006; Wesselingh 2007). In our case, the seed-set of free-pollinated flowers from non-manipulated and manipulated plants was comparable. This suggested that: (i) the effect of within-plant reallocation processes promoted by pollen supplementation was negligible, and (ii) the presence of bags on experimental plants did not influence pollinators.

Seed-set comparison between naturally and experimentally pollinated flowers suggested a significant pollen limitation in the studied population. Pollen limitation is a frequent consequence of human disturbance (Eckert *et al.* 2010; Brys and Jacquemyn 2012), but in our case it seemed linked to the variations of insect activity occurred over the studied ecological gradient. In addition, as common in *Dianthus* (Jennersten 1987; Collin and Shykoff 2003; Gargano *et al.* 2009), *D. balbisii* showed a little ability for autonomous self-fertilization, and this can have favored the rise of pollen limitation. Indeed, functional limitation for self-fertilization limit the chances of reproductive assurance (Kalisz *et al.* 2004). Anyway, the extent of pollen limitation varied greatly in our system, being higher at the gradient extremes. Variations in insect assemblages were demonstrated to generate spatial patterns of pollen limitation (Gómez *et al.* 2010). Accordingly, our data evidenced that spatial structuring of pollen limitation can occur at very small scale, following the ecological gradients typical of transition habitats. Although pollinators are essential to allow pollen transfer (Ashman *et al.* 2004), herbivory can also influence pollen delivery rates. Florivory can reduce the pollen available to pollinators (Hargreaves *et al.* 2009; Harder and Aizen 2010), or limit the efficiency of plant–pollinator interactions (Penet *et al.* 2009; Sõber *et al.* 2010; Lucas-Barbosa *et al.* 2011, 2016). Accordingly, the variations of both pollinators and herbivores contributed to model patterns of pollen limitation over the investigated gradient.

Pollen limitation also involves qualitative components of plant reproduction (i.e. offspring viability), which depend on the features of transferred pollen (Wilcock and Neiland 2002; Amat *et al.* 2011; Arceo-Gomez and Ashman 2014). The interplay between the structuring of plant populations and insect assemblages can affect the quality of pollen delivery (i.e. cross- vs. self-pollen) and, then, the viability in the subsequent offspring (Zorn-Arnold and Howe 2007; Le Cadre *et al.* 2008). In our study system, viability differed between progenies from hand-crosspollination and free pollination at low and high *LI* (Fig. 5B, C). Although *Dianthus* species are fully self-compatible, the presence of protandry and male-sterility promotes high outcrossing rates in their populations (Collin and Shykoff 2003). The consequent genetic load can result in severe inbreeding depression under enhanced selfing rates (Collin *et al.* 2009; Gargano *et al.* 2009, 2011, 2015). Therefore, the observed fitness variations can result from local variations in relative amount of cross- and self-pollinations. At intermediate *LI*, the fitness of naturally produced offspring was roughly equidistant from forced crossing and forced selfing (Fig. 5B, C). This would be compatible with the mixture of self- and cross-pollination typically occurring in plant populations (Wilcock and Neiland 2002). Instead, toward the gradient extremes the fitness of progenies from free-pollinated flowers declined, approaching the values detected after forced selfing (Fig. 5B, C). This suggested increased selfing rates. The accumulation of flowers in hermaphrodite phase on individuals, and the multiple within-plant visits due to *Bombylius* could have promoted inbreeding at low *LI*. Indeed, the presence of many open flowers on a single plant diminishes the effectiveness of dichogamy in preventing self-fertilization via geitonogamy (Petterson 1992; Hidalgo and Hubera 2001). Such a limitation can be exacerbated if the prevalent pollinators travel over short-distance (Zorn-Arnold and Howe 2007). Instead, herbivory could have enhanced selfing rates at high *LI*. Indeed, by reducing flower display and plant attractiveness, massive florivory can increases the chances of self-fertilization due to sporadic pollinator visits (Penet *et al.* 2009; Sõber *et al.* 2010). Then, the observed patterns of offspring viability can have reflected varying rates of inbreeding promoted by the interplay of: (i) habitat heterogeneity, (ii) insect fauna features (i.e. abundance, composition, and behaviour), and (iii) modality of pollen transfer.

## Conclusions

Our work evidences how the ecological heterogeneity found at forest edges can generate small-scale patterns of relative insect abundance that are the premise for local variations in *PBI*s and plant fitness. Shifts in both mutualistic and antagonist insects can influence *PBI*s across such habitat gradients, contributing to generate quantitative and qualitative patterns of plant fitness. Such patterns reveal that in edge habitats plants can rely on a very small ecological space to optimize their reproduction. Therefore, the conservation of edge-dependent species requires the preservation of the habitat complexity typical of such transition contexts. Further work is needed to evaluate the effect on *PBI*s of temporal habitat dynamics involving these peculiar ecological contexts.

## Supporting Information

The following additional information is available in the online version of this article—

**Table S1.** List and frequency of insects visiting flowers of *D. balbisii* during the observation trials, and their putative role in the context of the biotic interactions involving the study plant.

## Sources of Funding

D.G. was supported by Ministero dell’Istruzione, dell’Università e della Ricerca (funding source ex MURST 60 %).

## Contributions by the Authors

D.G. originally formulated the idea, and developed methodology; D.G. and L.B. conducted fieldwork, D.G. and G.F. performed statistical analyses; D.G., G.F. and L.B. wrote the manuscript.

## Conflict of Interest Statement

None declared.

## Supplementary Material

Supplementary Data

## References

[plx031-B1] Aguilar R, Ashworth L, Galetto L, Aizen MA. 2006 Plant reproductive susceptibility to habitat fragmentation: review and synthesis through a meta-analysis. Ecology Letters 9:968–980. 16913941 10.1111/j.1461-0248.2006.00927.x

[plx031-B2] Amat ME, Vargas P, Gomez JM. 2011 Pollen quality limitation in the Iberian critically-endangered genus *Pseudomisopates* (Antirrhinaceae). Plant Ecology 212:1069–1078.

[plx031-B3] Arceo-Gomez G, Ashman TL. 2014 Local co-flowering community diversity influences pollen receipt, female reproductive success and mediates selection on flower longevity in *Mimulus guttatus*. The American Naturalist 183:E50–E63. 10.1086/674358 24464206

[plx031-B4] Ashman T-L, Knight TM, Steets JA, Amarasekare P, Burd M, Campbell R, Dudash MR, Mazer SJ, Johnston M, Mitchell RJ, Morgan MT, Wilson WG. 2004 Pollen limitation of plant reproduction: ecological and evolutionary causes and consequences. Ecology 85:2408–2421.

[plx031-B5] Ashman T-L, Penet L. 2007 Direct and indirect effects of a sex-biased antagonist on male and female fertility: consequences for reproductive trait evolution in a gender-dimorphic plant. The American Naturalist 169:595–608. 10.1086/513150 17427131

[plx031-B6] Bhattacharya M, Primack RB, Gerwein J. 2003 Are roads and railroads barriers to bumblebee movement in a temperate suburban area?. Biological Conservation 109:37–45.

[plx031-B7] Block D, Werdenberg N, Erhardt A. 2006 Pollination crisis in the butterfly-pollinated wild carnation *Dianthus carthusianorum*?. New Phytologist 169:699–706. 16441751 10.1111/j.1469-8137.2006.01653.x

[plx031-B8] Brys R, Jacquemyn H. 2012 Effects of human-mediated pollinator impoverishment on floral traits and mating patterns in a short-lived herb: an experimental approach. Functional Ecology 26:189–197.

[plx031-B9] Cariveau D, Irwin RE, Brody AK, Garcia-Mayeya LS, von der Ohe A. 2004 Direct and indirect effects of pollinators and seed predators to selection on plant and flower traits. Oikos 104:15–26.

[plx031-B10] Caruso CM, Peterson B, Ridley C. 2003 Natural selection on floral traits of *Lobelia* (Lobeliaceae): spatial and temporal variation. American Journal of Botany 90:1333–1340. 21659233 10.3732/ajb.90.9.1333

[plx031-B11] Chacón P, Armesto JJ. 2006 Do carbon-based defences reduce foliar damage? Habitat-related effects on tree seedling performance in a temperate rainforest of Chlloé Island, Chile. Oecologia 146:555–565. 16170562 10.1007/s00442-005-0244-8

[plx031-B12] Chalcoff VR, Aizen MA, Ezcurra C. 2012 Erosion of a pollination mutualism along an environmental gradient in a south Andean treelet, *Embothrium coccineum* (Proteaceae). Oikos 121:471–480.

[plx031-B13] Collin CL, Shykoff JA. 2003 Outcrossing rates in the gynomoecious-gynodioecious species *Dianthus sylvestris* (Caryophyllaceae). American Journal of Botany 90:579–585. 21659152 10.3732/ajb.90.4.579

[plx031-B14] Collin CL, Penet L, Shykoff JA. 2009 Early inbreeding depression in the sexually polymorphic plant *Dianthus sylvestris* (Caryophyllaceae): effects of selfing and biparental inbreeding among sex morphs. American Journal of Botany 96:2279–2287. 21622343 10.3732/ajb.0900073

[plx031-B15] Colwell RK. 2013 EstimateS: Statistical estimation of species richness and shared species from samples. Version 9. Persistent URL: purl.oclc.org/estimates.

[plx031-B16] Colwell RK, Chao A, Gotelli NJ, Lin S-Y, Mao CX, Chazdon RL, Longino JT. 2012 Models and estimators linking individual-based and sample-based rarefaction, extrapolation and comparison of assemblages. Journal of Plant Ecology 5:3–21.

[plx031-B17] Dovčiak M, Brown J. 2014 Secondary edge effects in regenerating forest landscapes: vegetation and microclimate patterns and their implications for management and conservation. New Forests 45:733–744.

[plx031-B64] Eckert CG, Kalisz S., Geber MA, Sargent R., Elle E., Cheptou P-O, Goodwillie C., Johnston MO, Kelly JK, Moeller DA, Porcher E, Ree RH, Vallejo-Marin M., Winn A. 2010 Plant mating systems in a changing world. Trends in Ecology and Evolution 25:35–43. 19683360 10.1016/j.tree.2009.06.013

[plx031-B18] Erhardt A. 1988 Pollination and reproduction in *Dianthus**silvester* Wulf In: Cresti M, Gori P, Pacini E, eds. Sexual reproduction in higher plants. Berlin: Springer, 361–356.

[plx031-B19] Falcucci A, Maiorano L, Boitani L. 2007 Changes in land-use/cover patterns in Italy and their implications for biodiversity conservation. Landscape Ecology 22:617–631.

[plx031-B20] Forman RTT. 1995 Land mosaics. The ecology of landscapes and regions. Cambridge: Cambridge University Press.

[plx031-B21] Gargano D, Gullo T, Bernardo L. 2009 Do inefficient selfing and inbreeding depression challenge the persistence of the rare *Dianthus guliae* Janka (Caryophyllaceae)? Influence of reproductive traits on a plant's proneness to extinction. Plant Species Biology 24:69–76.

[plx031-B22] Gargano D, Gullo T, Bernardo L. 2011 Fitness drivers in the threatened *Dianthus guliae* Janka (Caryophyllaceae): disentangling effects of growth context, maternal influence and inbreeding depression. Plant Biology 13 (S1):96–103. 21134092 10.1111/j.1438-8677.2010.00392.x

[plx031-B23] Gargano D, Pellegrino G, Bernardo L. 2015 Genetic and fitness consequences of interpopulation mating in *Dianthus guliae* Janka: conservation implications for severely depleted and isolated plant populations. Conservation Genetics 16:1127–1138.

[plx031-B24] Ghazoul J. 2005 Buzziness as usual? Questioning the global pollination crisis. Trends in Ecology and Evolution 20:367–373. 16701398 10.1016/j.tree.2005.04.026

[plx031-B25] Gómez JM. 2003 Herbivory reduces strength of pollinator-mediated selection in the mediterranean herb *Erysimum mediohispanicum*: consequences for plant specialization. The American Naturalist 162:242–256. 10.1086/376574 12858267

[plx031-B26] Gómez JM. 2005 Non-additive effects of herbivores and pollination on *Erysimum mediohispanicum* (Cruciferae) fitness. Oecologia 143:412–418. 15678331 10.1007/s00442-004-1809-7

[plx031-B27] Gómez JM. 2008 Sequential conflicting selection due to multispecific interactions triggers evolutionary trade-offs in a monocarcpic herb. Evolution 62:668–679. 18182075 10.1111/j.1558-5646.2007.00312.x

[plx031-B28] Gómez JM, Abdelaziz M, Lorite J, Muñoz-Pajares AJ, Perfectti F. 2010 Changes in pollinator fauna cause spatial variation in pollen limitation. Journal of Ecology 98:1243–1252.

[plx031-B29] Hadley AS, Betts MG. 2012 The effects of landscape fragmentation on pollination dynamics: absence of evidence not evidence of absence. Biological Reviews 87:526–544. 22098594 10.1111/j.1469-185X.2011.00205.x

[plx031-B30] Harder LD, Aizen MA. 2010 Floral adaptation and diversification under pollen limitation. Philosophical Transactions of the Royal Society B 365:529–543. 10.1098/rstb.2009.0226 PMC2838256 20047878

[plx031-B31] Harder LD, Barrett SCH. 2006 The ecology and evolution of flowers. Oxford: Oxford University Press.

[plx031-B32] Hargreaves AL, Harder LD, Johnson SD. 2009 Consumptive emasculation: the ecological and evolutionary consequences of pollen theft. Biological Review 84:259–276. 10.1111/j.1469-185X.2008.00074.x 19382932

[plx031-B33] Hidalgo PJ, Hubera JL. 2001 Inbreeding depression in *Rosmarinus officinalis* L. International Journal of Developmental Biology 45:S43–S44.

[plx031-B34] Huang B-A, Sun Y-N, Yu X-H, Luo Y-B, Hutchings MJ, Tang S-Y. 2009 Impact of proximity to a pathway on orchid pollination success in Huanglong National Park, South West China. Biological Conservation 142:701–708.

[plx031-B35] Jennersten O. 1987 Pollination in *Dianthus deltoides* (Caryophyllaceae): effects of habitat fragmentation on visitation and seed set. Conservation Biology 2:359–366.

[plx031-B36] Joffre R, Rambal S. 1993 How tree cover influences the water balance in Mediterranean rangelands. Ecology 74:570–582.

[plx031-B37] Kastinger C, Weber A. 2001 Bee-flies (Bombylius spp., Bombyliidae, Diptera) and the pollination of flowers. Flora 196:3–25.

[plx031-B38] Knight TM, Steets JA, Ashman T-L. 2006 A quantitative synthesis of pollen supplementation experiments highlights the contribution of resource reallocation to estimates of pollen limitation. American Journal of Botany 93:271–277. 21646188 10.3732/ajb.93.2.271

[plx031-B39] Knight TM, Steets JA, Vamosi JC, Mazer SJ, Burd M, Campbell DR, Dudash MR, Johnston MO, Mitchell RJ, Ashman T-L. 2005 Pollen limitation of plant reproduction: pattern and process. Annual Review of Ecology, Evolution, and Systematics 36:467–497.

[plx031-B40] Le Cadre S, Tully T, Mazer SJ, Ferdy J-B, Moret J, Machon N. 2008 Allee effects within small populations of *Aconitum napellus* ssp. *lusitanicum*, a protected subspecies in northern France. New Phytologist 179:1171–1182. 18557816 10.1111/j.1469-8137.2008.02529.x

[plx031-B41] Lennartsson Y. 2002 Extinction thresholds and disrupted plant–pollinator interaction in fragmented plant populations. Ecology 83:3060–3072.

[plx031-B42] López-Barrera F, Armesto JJ, Williams-Linera G. 2007 Fragmentation and edge effect on plant–animal interactions, ecological processes and biodiversity In: Newton AC, ed. Biodiversity loss and conservation in fragmented forest landscapes: the forests of montane Mexico and temperate South America. Egham: CABI, 69–101.

[plx031-B43] Lucas-Barbosa D. 2016 Integrating studies on plant–pollinator and plant–herbivore interactions. Trends in Plant Science 21:125–133. 26598297 10.1016/j.tplants.2015.10.013

[plx031-B44] Lucas-Barbosa D, van Loon JJA, Dicke M. 2011 The effects of herbivore-induced plant volatiles on interactions between plants and flower-visiting insects. Phytochemistry 72:1647–1654. 21497866 10.1016/j.phytochem.2011.03.013

[plx031-B45] Lucas-Barbosa D, Sun P, Hakman A, van Beek TA, van Loon JJA, Dicke M. 2016 Visual and odour cues: plant responses to pollination and herbivory affect the behavior of flowers visitors. Functional Ecology 30:431–441.

[plx031-B46] McCall AC, Irwin RE. 2006 Florivory: the intersection of pollination and herbivory. Ecology Letters 9:1351–1365. 17118009 10.1111/j.1461-0248.2006.00975.x

[plx031-B47] Ollerton J, Winfree R, Tarrant S. 2011 How many flowering plants are pollinated by animals? Oikos 120:321–326.

[plx031-B48] Pauw A, Hawkins JA. 2011 Reconstruction of historical pollination rates reveals linked declines of pollinators and plants. Oikos 120:344–349.

[plx031-B49] Penet L, Collin CL, Ashman T-L. 2009 Florivory increases selfing: an experimental study in the wild strawberry, *Fragaria virginiana*. Plant Biology 11:38–45. 19121112 10.1111/j.1438-8677.2008.00141.x

[plx031-B50] Peruzzi L, Gargano D. 2006 *Dianthus ferrugineus* Mill. vs. *D. guliae* Janka: nomenclatural considerations on the Italian yellow carnation. Taxon 55:781–784.

[plx031-B51] Petterson MW. 1992 Advantages of being a specialist female in gynodioecious *Silene vulgaris* s.l. (Caryophyllaceae). American Journal of Botany 79:1389–1395.

[plx031-B52] Pignatti S. 1982 Flora d’Italia 1. Bologna: Edagricole.

[plx031-B53] Ries L, Fletcher RJ Jr, Battin J, Sisk TD. 2004 Ecological responses to habitat edges: mechanisms, models, and variability explained. Annual Review of Ecology, Evolution, and Systematics 35:491–522.

[plx031-B54] Risser PG. 1995 The status of the science examining ecotones. BioScience 45:318–325.

[plx031-B55] Sarlov-Herlin I. 2001 Approaches to forest edges as dynamics structures and functional concepts. Landscape Research 26:27–43.

[plx031-B56] Schoonhoven LM, van Loon JJA, Dicke M. 2005 Insect-plant biology. Oxford: Oxford University Press.

[plx031-B57] Sõber V, Moora M, Teder T. 2010 Florivores decrease pollinator visitation in a self-incompatible plant. Basic and Applied Ecology 11:669–675.

[plx031-B58] Soper Gorden NL, Adler LS. 2016 Florivory shapes both leaf and floral interactions. Ecosphere 7:e01326.

[plx031-B59] Totland Ø. 2001 Environment-dependent pollen limitation and selection on floral traits in an alpine species. Ecology 82:2233–2244.

[plx031-B60] Verboven HAF, Aertsen W, Brys R, Hermy M. 2014 Pollination and seed set of an obligatory outcrossing plant in a urban-peri-urban gradient. Perspectives in Plant Ecology, Evolution, and Systematics 16:121–131.

[plx031-B61] Wesselingh RA. 2007 Pollen limitation meets resource allocation: towards a comprehensive methodology. New Phytologist 174:26–37. 17335494 10.1111/j.1469-8137.2007.01997.x

[plx031-B62] Wilcock C, Neiland R. 2002 Pollination failure in plants: why it happens and when it matters. Trends in Plant Science 7:270–277. 12049924 10.1016/s1360-1385(02)02258-6

[plx031-B63] Zorn-Arnold B, Howe HF. 2007 Density and seed set in a self-compatible forb, *Penstemon digitalis* (Plantaginaceae), with multiple pollinators. American Journal of Botany 94:1594–1602. 21636358 10.3732/ajb.94.10.1594

