# Supplementary material for: Local shifts in floral biotic interactions in habitat edges and their effect on quantity and quality of plant offspring
Source: AoB Plants. 2017 Jul 26;9(4):plx031. doi: 10.1093/aobpla/plx031 (PMC5534021; doi:10.1093/aobpla/plx031)
Supplement: Supplementary Data [file plx031_Supp.doc]

| **Table S1.** List and frequency of insects visiting flowers of *D. balbisii* during the observation trials, and their putative role in the context of the biotic interactions involving the study plant. | | | |
| --- | --- | --- | --- |
|
|  |  |  |  |
| **Visitor** | **Code** | **No. of visits** | **Prevalent function in *PBI*s** |
| *Thymelicus sylvestris* | THY | 1736 | Pollinator |
| *Bombylius major* | BOM | 552 | Pollinator |
| *Macroglossum stellatarum* | OTH | 28 | Pollinator |
| *Papilio macaon* | OTH | 116 | Pollinator |
| *Polyommatus* sp. | OTH | 12 | Pollinator |
| *Pieris* sp. | OTH | 29 | Pollinator |
| *Sibinia viscariae* | HER | 512 | Herbivore/pre-dispersal predator |
| Other Curculionidae | HER | 164 | Herbivore |
| Formicidae | HER | 115 | Pollen robber |
|  |  |  |  |
